# Serum-borne factors alter cerebrovascular endothelial microRNA expression following particulate matter exposure near an abandoned uranium mine on the Navajo Nation

**DOI:** 10.1186/s12989-020-00361-3

**Published:** 2020-07-01

**Authors:** Bethany Sanchez, Xixi Zhou, Amy S. Gardiner, Guy Herbert, Selita Lucas, Masako Morishita, James G. Wagner, Ryan Lewandowski, Jack R. Harkema, Chris Shuey, Matthew J. Campen, Katherine E. Zychowski

**Affiliations:** 1 Department of Pharmaceutical Sciences, University of New Mexico-Health Sciences Center, Albuquerque, NM 87131 USA; 2 Department of Cell Biology and Physiology, University of New Mexico-Health Sciences Center, Albuquerque, NM 87131 USA; 3 Department of Family Medicine, College of Human Medicine, Michigan State University, East Lansing, MI USA; 4 Department of Pathobiology and Diagnostic Investigation, College of Veterinary Medicine, Michigan State University, East Lansing, MI USA; 5 Southwest Research and Information Center, Albuquerque, NM USA; 6 College of Nursing, MSC09 53601 University of New Mexico-Health Sciences Center, Albuquerque, NM 87131 USA

**Keywords:** Endothelial, PM_2.5_, microRNAs, Serum, Mmu-*let-7a*, Blood-brain barrier, Uranium

## Abstract

**Background:**

Commercial uranium mining on the Navajo Nation has subjected communities on tribal lands in the Southwestern United States to exposures from residual environmental contamination. Vascular health effects from these ongoing exposures are an active area of study. There is an association between residential mine-site proximity and circulating biomarkers in residents, however, the contribution of mine-site derived wind-blown dusts on vascular and other health outcomes is unknown. To assess neurovascular effects of mine-site derived dusts, we exposed mice using a novel exposure paradigm, the AirCARE1 mobile inhalation laboratory, located 2 km from an abandoned uranium mine, Claim 28 in Blue Gap Tachee, AZ. Mice were exposed to filtered air (FA) (*n* = 6) or concentrated ambient particulate matter (CAPs) (*n* = 5) for 2 wks for 4 h per day.

**Results:**

To assess miRNA differential expression in cultured mouse cerebrovascular cells following particulate matter (PM) exposure (average: 96.6 ± 60.4 μg/m^3^ for all 4 h exposures), the serum cumulative inflammatory potential (SCIP) assay was employed. MiRNA sequencing was then performed in cultured mouse cerebrovascular endothelial cells (mCECs) to evaluate transcriptional changes. Results indicated 27 highly differentially expressed (*p* < 0.01) murine miRNAs, as measured in the SCIP assay. Gene ontology (GO) pathway analysis revealed notable alterations in GO enrichment related to the cytoplasm, protein binding and the cytosol, while significant KEGG pathways involved pathways in cancer, axon guidance and Wnt signaling. Expression of these 27 identified, differentially expressed murine miRNAs were then evaluated in the serum. Nine of these miRNAs (~ 30%) were significantly altered in the serum and 8 of those miRNAs demonstrated the same directional change (either upregulation or downregulation) as cellular miRNAs, as measured in the SCIP assay. Significantly upregulated miRNAs in the CAPs exposure group included miRNAs in the *let-7a* family. Overexpression of *mmu-let-7a* via transfection experiments, suggested that this miRNA may mediate mCEC barrier integrity following dust exposure.

**Conclusions:**

Our data suggest that mCEC miRNAs as measured in the SCIP assay show similarity to serum-borne miRNAs, as approximately 30% of highly differentially expressed cellular miRNAs in the SCIP assay were also found in the serum. While translocation of miRNAs via exosomes or an alternative mechanism is certainly possible, other yet-to-be-identified factors in the serum may be responsible for significant miRNA differential expression in endothelium following inhaled exposures. Additionally, the most highly upregulated murine miRNAs in the CAPs exposure group were in the *let-7a* family. These miRNAs play a prominent role in cell growth and differentiation and based on our transfection experiments, *mmu-let-7a* may contribute to cerebrovascular mCEC alterations following inhaled dust exposure.

## Introduction

Commercial uranium mining was active on the Navajo Nation from the 1940s–1980s during the Cold War era [1–3]. As a result of commercial uranium mining and insufficient remediation, many tribal communities located in the Southwestern United States are exposed to varying degrees of contaminant metal mixtures arising from abandoned uranium mines (AUMs) [4–6]. Exposure to metals through drinking water has been linked to adverse health outcomes [7–10], and residential proximity to AUMs is associated with an increase in circulating inflammatory biomarkers [11]. However, the contribution of wind-blown dust exposure on vascular and other health outcomes remains unclear. Moreover, there is a lack of consensus in the literature regarding how to evaluate vascular outcomes following PM_2.5_ exposure [12, 13]. Classical biomarkers of ingestion-related metals-exposure typically included assessment of metal concentration in the urine and blood [14]. However, metal levels detected in these biofluids do not necessarily translate to a specific pathological outcome, therefore assessing alternative molecular biomarkers of exposure may be warranted.

Residential proximity to AUMs is significantly associated with cumulative circulating inflammatory potential in the serum of local residents, as determined by an ex vivo mCEC cell bioassay, the serum cumulative inflammatory potential (SCIP) assay, developed in our laboratory [6, 11, 15, 16]. The SCIP assay is used to assess mCEC cellular mRNA and protein responses to circulating serum-borne ligands following inhaled toxicant exposure or environmental insult [15–20]. This assay has detected variations of mRNA and protein expression between control and exposed groups. Differential expression of mCEC cellular microRNAs (miRNAs) as measured in the SCIP assay have yet to be explored.

MiRNAs are small non-coding RNAs responsible for silencing gene expression. They are roughly 22 base pairs long and originate in the nucleus of the cell, but can also be found in the extracellular environment in various biological fluids including serum [21], interstitial fluid [22–24], and urine [25–27]. More recently, circulating miRNAs have been utilized as biomarkers of environmental and toxicological exposure [21, 28], with documented associations between metal exposure and circulating miRNAs [29–31]. MiRNAs also play a key role in regulating neuroinflammation and the blood-brain barrier (BBB) [32, 33]. Recent research from our group indicates that inhaled pollutants prompt a systemic inflammatory response resulting in mCEC dysfunction, a key feature of BBB disruption [34–36]. Compromising the BBB via inhaled toxicant exposure may potentiate preexisting vulnerability to a neurovascular event such as a stroke or aneurysm rupture [37, 38]. Additionally, air pollution has been implicated in neuroinflammation and diseases of the central nervous system [39–41]. While the molecular mechanisms underlying air pollution-induced neurological outcomes are unclear, one current hypothesis is that peptide fragments shed from the lung following inhaled pollutant exposures induce mCEC dysfunction via scavenger receptors [17, 34, 42]. Recent studies, using the SCIP assay following pulmonary exposure to multiwalled nanomaterials, highlight extensive peptidomic changes to the serum composition that align with changes in the serum bioactivity [43]. Nanotube exposure induces elevations in circulating peptides that appear to arise from endogenous proteins, with the hypothesis that matrix metalloproteinase (MMP) activation in the lung may trigger the generation of circulating fragmented peptides. These peptides, in turn, have a retained cumulative bioactivity on the endothelium that may impair barrier integrity and vasodilatory pathways.

In an effort to more thoroughly characterize the burden of wind-blown dust-driven health effects in this region, more specifically, neurovascular dysfunction, we exposed mice to ambient on-site PM using a mobile air research laboratory, AirCARE1 near an AUM, Claim 28, in the central region of Navajo Nation. MiRNA sequencing was performed in mCECs treated with serum from exposed mice (SCIP assay) to evaluate transcriptional changes and the most significantly altered murine cellular miRNAs were then assessed in the serum. One highly-upregulated miRNA in the CAPs exposure group (*mmu-let-7a*), was transfected in cultured mCECs in vitro using the SCIP assay and endothelial barrier integrity endpoints were assessed. In this exploratory study, we hypothesized that exposure to CAPs induces circulating factors that drive differential mCEC miRNA differential expression, as characterized in the SCIP assay. To our knowledge, this is the first assessment of mCEC miRNA alterations, as a result of serum-borne factors from mice exposed to FA and CAPs.

## Methods and materials

### Study site

Deployment of AirCARE1 was executed in Blue Gap Tachee, AZ, roughly 2 km away from the Claim 28 mine site on the Navajo Nation Reservation. Wind speed and direction were collected at 15-min intervals using meteorological stations (WindLog, RainWise, Inc., Trenton, ME, USA) placed on the roof of AirCARE1 and on the mesa ridge of the mine site during the course of the exposure.

### AirCARE1 inhalation laboratory exposure paradigm

Michigan State University’s AirCARE1, a mobile inhalation toxicology laboratory constructed within a renovated 53′ semitrailer, utilizes a Harvard fine-type particle concentrator to expose animals to ambient on-site PM in tandem with real-time PM monitoring. The fine particle concentrator performance and the exposure systems in AirCARE1 have been described in detail previously [44, 45]. Exposures were executed 4 h per day for 15 consecutive days in November/December 2017. Six-eight wk. old C57BL/6 male mice (Taconic, Rensselaer, NY) were randomly assigned to either filtered air (FA, *n* = 6) or particulate matter < 2.5 m (CAPs, *n* = 5) exposure. Immediately after the mice were loaded into the chambers, the doors were sealed. Animals were placed in Hinners stainless steel, whole-body exposure chambers to acclimate roughly 1 h prior to the start of the exposure. Exposures started at 9 AM and continued until 1 PM. Monitoring ensured a flow rate of 50 L/min at 0.94–0.95 atm. The PM mass was collected on 47-mm Teflon (PTFE) filters (Gelman Science) in Teflon/Teflon-coated filter packs attached to the side of the exposure chamber. A Sidepak Personal Aerosol Monitor (TSI, Shoreview, MN, USA) was used to measure real-time PM in the exposure chambers; final gravimetric concentrations derived from filter weights (as described below) were used as the actual mass concentration assessment. The pressure in the FA chamber was adjusted to the same level in the CAPs chamber (0.94–0.95 atm). HEPA-filtered room air (maintained at 21 °C and 60 ± 10% relative humidity) was supplied to the control chamber at 50 L/min. Meteorological endpoints including temperature, wind speed and direction were all collected in real-time. Following the end of exposures, the chamber was switched back to FA and the pressure in both exposure chambers was raised to atmospheric pressure. Food and water were provided following the exposures, as per IACUC protocol. Mice were transported to the University of New Mexico following the end of exposures and euthanized for tissue collection and downstream analyses 24 h later.

### Particulate elemental composition analysis

The Teflon filter samples were shipped to the Michigan State University Exposure Science Laboratory and subsequently processed and analyzed in their Class 100 clean room. The gravimetric mass concentrations were determined from the filters using a microbalance (XP6; Mettler-Toledo) in a temperature- and humidity-controlled clean room as described in the EPA Federal Reference Method. Following the gravimetric analysis, the Teflon filters were analyzed for trace metals. The Teflon filters were placed in 15 mL centrifuge tubes and moistened with 150 L of ethanol before extraction in 10 mL of 1% HNO_3_. The 1% HNO_3_ solution was then sonicated in an ultrasonic bath for 48 h and used to passively digest the filter for 2 wks. Sample extracts were then analyzed for selected trace elements using high-resolution inductively coupled plasma mass spectrometry (ICP-MS, ELEMENT2, Thermo Finnigan, San Jose, CA). Commercially available multi-element standards were used to create a standard curve in a 1% HNO_3_ solution and the matrix was matched with the samples. The *R*^2^ value was determined for each element via ELEMENT software. Data including daily QA/QC measurements such as field blanks, acid blanks, laboratory blanks and replicate analyses were matched with the samples. Standards were analyzed as samples to evaluate instrument stability and the National Institute of Standards and Technology (NIST, Gaithersburg, MD) Standard Reference Material 1640A was used as a quality control standard.

### Bronchoalveolar lavage fluid analysis

Mice were euthanized 24 h post-CAPs exposure. Mice were sedated with 5% isoflurane at a rate of 5 L/min until unresponsive, and subsequently subjected to cardiac puncture and exsanguination. Bronchoalveolar lavage fluid (BALF) analysis was performed following euthanasia by puncturing the trachea and intubating each mouse using a 20-gauge cannula. One mL of PBS was then injected into the lungs and pulled out following inflation. Total BALF cells, macrophages and neutrophils were counted using a hemocytometer and stained with Trypan Blue (Thermo Fisher Scientific, Waltham, MA, USA) to determine viability. BALF was then centrifuged at 500 x g for 10 min at 4 °C and supernatants were collected and analyzed. BALF supernatant total protein was assessed using a bicinchoninic acid (BCA) assay [46] and read at an absorbance of 595 nm using a Tecan plate reader (Tecan, Mannedorf, Switzerland).

### Cell culture

Mouse cerebrovascular endothelial cells were grown to approximately 80% confluence as per the manufacturer’s instructions (Lonza, Basel, Switzerland). Flasks were coated with poly-l-lysine prior to plating. Basal complete media was replaced every other day and cells were passaged once per week.

### Serum cumulative inflammatory potential (SCIP) bioassay

Mouse cerebrovascular endothelial cells (mCECs) (Lonza, Allendale, NJ) were seeded in a 24-well plate and grown to confluence in complete media (Lonza). In total, 11 samples were analyzed with 6 biological replicates in the FA group and 5 biological replicates in the CAPs group. Cells were serum-starved for 24 h prior to exposure, to synchronize all of the cells to the same cell cycle phase. Confluent mCECs were incubated with serum from CAPs-exposed mice and FA-exposed mice (20 μl serum in 450 μl of serum-free media). Samples were incubated at 37 °C for 4 h. RNA was extracted from the cells using a commercial kit (RNeasy, Qiagen, Germantown, Maryland) according to the manufacturer’s instructions. This RNA was then aliquoted and frozen at − 80°C.

### MicroRNA sequencing

Total RNA, including small RNAs, was extracted using a commercial extraction kit from Total RNA Purification Kit from Norgen Biotek Corp (Thorold, ON, Canada) in cultured mCECs in the SCIP assay. RNA quality and quantity were analyzed using a Bioanalyzer 2100 (Agilent, CA, USA) to ensure a RIN number > 7.0. A small RNA library was prepared using TruSeq Small RNA Sample Prep Kit (Illumina, San Diego, USA) using 1 g of total RNA. Fifty bp single-end sequencing on an Illumina HiSeq 2500 at LC Sciences (Hangzhou, China) was performed as per the manufacturer’s instructions.

### Bioinformatics analysis

Adapter dimers, junk, low complexity, common RNA families including rRNA, tRNA, snRNA and snoRNA and repeats were subjected to an in-house program, ACGT101-miR (LC Sciences, Houston, Texas, USA). Unique sequences 18–26 nucleotides in length were mapped to specific species precursors in miRbase 22.0 by BLAST search to identify known miRNAs and novel 3p- and 5p-derived miRNAs. The remaining sequences were mapped to selected species precursors (with the exclusion of specific species) in miRbase 22.0 via BLAST search. The mapped pre-miRNAs were then BLASTed against the specific species genomes to establish their genome locations. These were defined as known miRNAs. Secondary structure predictions were established by: 1) number of nucleotides in one bulge in stem (≤12), 2) number of base pairs in the stem region of the predicted hairpin (≥16), 3) cutoff of free energy (kCal/mol ≤ − 15), 4) length of hairpin (up and down stems + terminal loop ≥50), 5) length of hairpin loop (≤20), 6) number of nucleotides in one bulge in mature region ((≤8), 7) number of biased errors in one bulge in mature region (≤4), 8) number of biased bulges in mature region (≤2), 9) number of errors in mature region (≤7), 10) number of base pairs in the mature region of the predicted hairpin (≥12), and 11) percent of mature regions in stem (≥80). In total, 11 samples were analyzed with 6 biological replicates in the FA group and 5 biological replicates in the CAPs group.

### Target prediction of differentially expressed microRNAs

Predicted genes targeted by the most abundant miRNAs were assessed using two algorithms, TargetScan (http://www.targetscan.org/) and Miranda 3.3a (http://www.microrna.org/), to assess binding sites. Data predicted by these algorithms were combined and subsequent overlaps were calculated. The GO terms and KEGG pathways of abundant miRNAs and targets were also annotated. Significant GO terms were calculated using the following Hypergeometric equation described below:

$$ P=1-\sum \limits_{i=0}^{s-1}\frac{\left(\frac{B}{i}\right)\left(\frac{TB-B}{TS-i}\right)}{\left(\frac{TB}{TS}\right)} $$

Differential expression with 3 randomly selected miRNAs in the *p* < 0.01 group were validated using qPCR (data not shown).

### Circulating serum-borne microRNAs

Fireplex multiplexing technology (Abcam, Cambridge, MA) was used to quantify the most significantly altered (*p* < 0.01) serum miRNAs of murine origin identified following the miRNA sequencing experiment mentioned above. Briefly, samples were assayed in duplicates and 20 L of serum per replicate was hybridized to the Fireplex plate and captured by target-specific probes embedded in Firefly barcoded hydrogel particles for 60 min at room temperature. Oligonucleotide adapters were then ligated to each end of the hybridized miRNA as PCR priming sites and amplified for 60 min. Labeled miRNAs were then dehybridized from particles and underwent one-step RT-PCR with a biotinylated primer. PCR products were then rehybridized to the original Firefly particles and subsequently incubated with a fluorescent reporter for detection. Particles were then scanned using an EMD Millipore 8HT flow cytometer (Burlington, MA, USA). MiRNAs used for normalization included mmu-mir-425-5p_r-1, mmu-mir-744-5p_r-1, mmu-mir-16-5p. Cel-mir-70-3p was used as an exogenous control. Flow cytometry files were analyzed using Firefly Analysis Workbench (Abcam, Cambridge, MA).

### mRNA prediction and network analysis

The Bioconductor package *miRNAtap* was used to predict mRNA targets. Targets were aggregated from 5 prediction algorithms: DIANA [47], Miranda [48], PicTar [49], TargetScan [50], and miRDB [51]. mRNA targets found in at least 2 algorithms were considered valid. Then, a *ReactomePA* [52] package was used to analyze reactome pathway enrichment on predicted mRNA targets. The R script performing all analyses and a list of predicted mRNA targets are available at: https://github.com/Perl-R/190606-Uranium-miRNA.

### *mmu-let-7a* transfections

Mmu-let-7a mimic and let-7a negative control miRNA (Qiagen, Germantown, MD) were transfected into a monolayer of mCECs using the Applied Biosystems Electric Cell-substrate Impedance Sensing (ECIS) system at 10^5^ cells per well. Reverse transfection protocol was utilized as per the HiPerfect Transfections manufacturer’s instructions (Qiagen). After cells were grown to confluence, as determined by a plateau in transendothelial electrical resistance (TEER), serum was added from FA- and CAPs-exposed mice and grown for 45 h. TEER readings were captured in real-time every 10 min until the final endpoint.

### Statistics

MiRNA differential expression was based on normalized deep-sequencing counts and analyzed by selectively using Fisher’s exact test, Chi-squared nXn test, Student’s *t*-test or ANOVA, based on experimental design. The data heatmap where the individual z values (repeated) or Log_10_ (non-repeated) within a matrix are represented within the red-blue color scheme, as indicated in the heat-map. For the transfection studies, a one-way ANOVA followed by Tukey’s multiple comparisons, post-hoc test was performed.

## Results

### Particulate matter (PM) and wind characterization

**Fig. 1:**
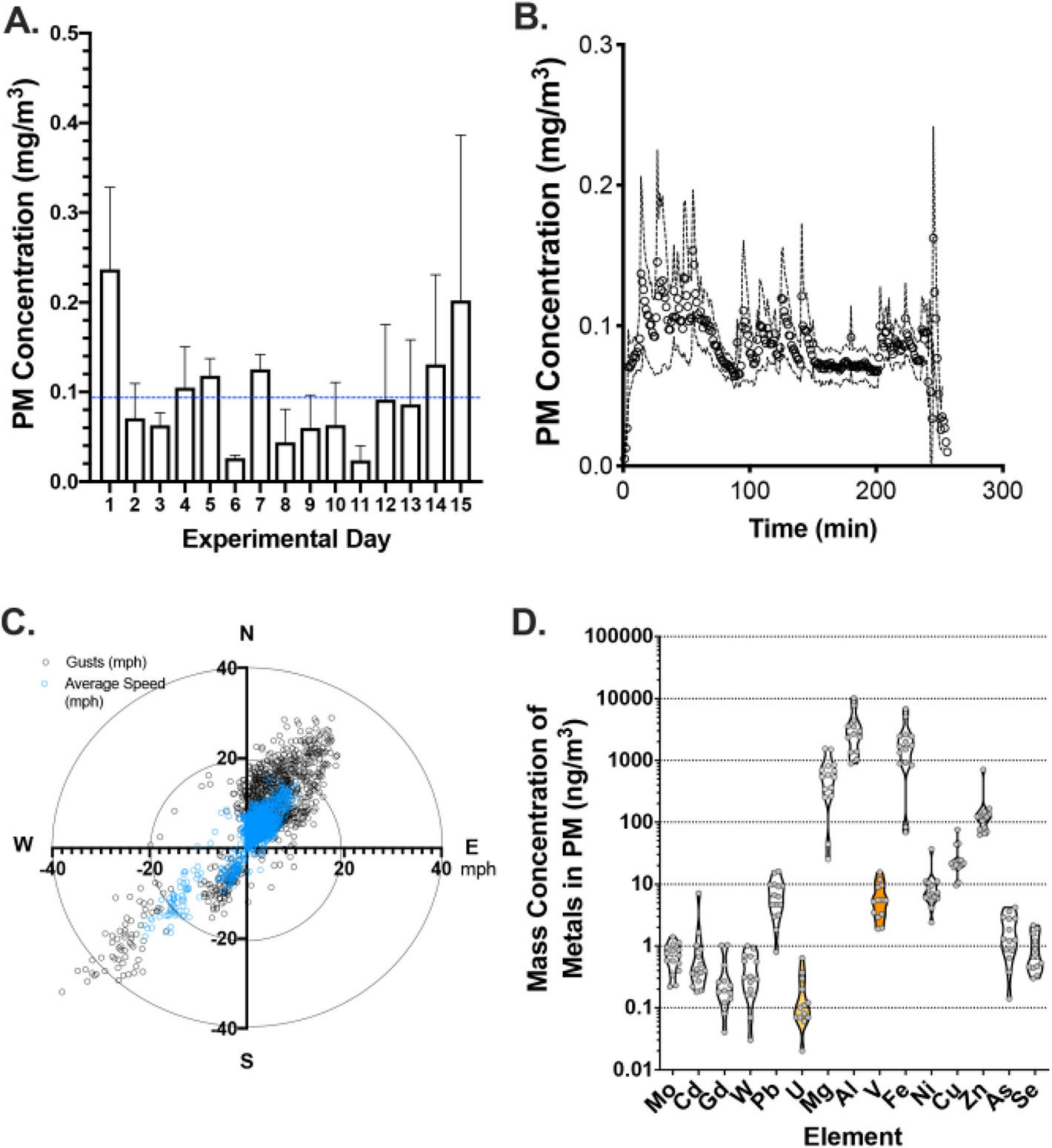
Exposures in Blue Gap Tachee, AZ. CAPs characterization data via analytical methods and AirCARE 1 wind exposure metrics. **a** CAPs concentration on each exposure day (mg/m^3^) **b** CAPs concentration (mg/m^3^) over the exposure time frame. The average CAPs concentration over 4 h was recorded at 96.6 ± 60.4 μg/m^3^ (mean ± SEM) **c** Wind speed as assessed at the exposure site. Wind generally blew from the southwest or northeast throughout the exposure period. **d** Mass concentration of metals in CAPs (mg/m^3^)

Concentrated particulate levels varied substantially from day-to-day during the 2-week period (Fig. 1a). The 4 h exposure in the mobile laboratory, however, showed a relatively consistent operation (Fig. 1b). Relative to the AirCARE1 location, the wind gusts most often blew from the southwest or northeast and varied throughout the exposure period (Fig. 1c). The average exposure for all 4 h periods was 96.6 ± 60.4 μg/m^3^. The average concentrations of key metals are shown in Fig. 1d. Metals detected in the filter samples included Mo, Cd, Gd, W, Pb, U, Mg, Al, V, Fe, Ni, Cu, Zn, As, and Se. While samples previously collected from the Claim 28 mine-site, showed the presence of both U and V (highlighted in the figure, Zychowski et al. 2018), our current results suggest that metals present in airborne CAPs included higher levels of Al and Fe.

### Bronchoalveolar lavage and number of cells

**Fig. 2:**
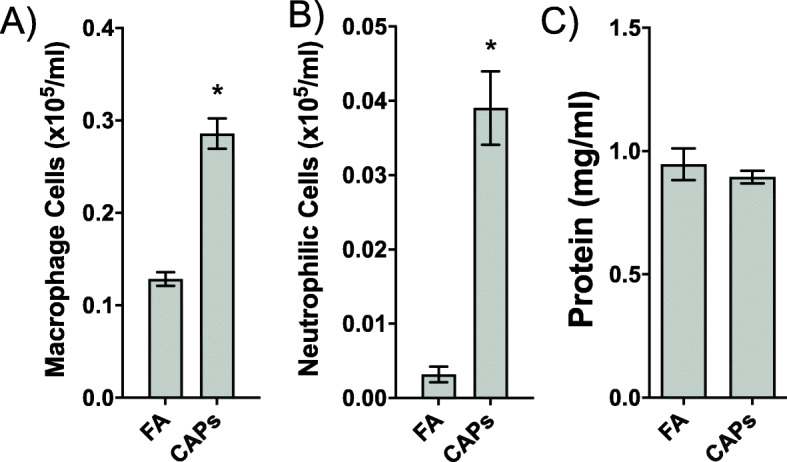
Bronchoalveolar lavage (BAL) fluid parameters. Following euthanasia of each mouse, BAL was collected via tracheal puncture and assessed for number of inflammatory cells for **a** macrophages (cells × 10^5^/mL) and **b** neutrophils (cells × 10^5^/mL) **c** protein (mg/mL). Macrophage and neutrophilic cell counts were significantly upregulated in the CAPs exposure group relative to FA exposure, based on a Student’s t-test (*p* < 0.05), indicating an inflammatory pulmonary response following CAPs exposure

Cellularity was significantly altered between FA and CAPs exposure. Macrophage counts and neutrophils were significantly increased in the CAPs exposed group compared to FA (Fig. 2a, b). However, protein (mg/mL), a marker of pulmonary endothelial barrier integrity, was not significantly different between exposure groups (Fig. 2c).

### MicroRNA differential expression

**Fig. 3:**
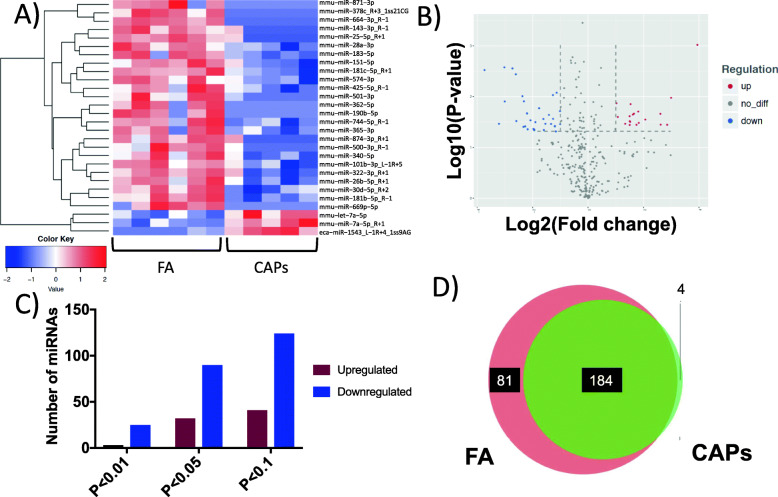
MicroRNA differential expression. Differential miRNA expression between FA and CAPs, as measured in the SCIP assay **a** Heatmap of significantly differentially expressed (*p* < 0.01) miRNAs. The heatmap is a graphical representation of individual values in a matrix represented in a red (upregulated) or blue (downregulated) color scheme **b** volcano plot indicating significance (y-axis) and log_2_ fold-change (x-axis) **c** number of upregulated and downregulated miRNAs **d** FA vs. CAPs miRNAs Venn-diagram

There were 27 highly significant (*p* < 0.01) differentially expressed miRNAs of murine origin between FA- and CAPs-exposed groups (Fig. 3a, b) in mCECs, as measured in the SCIP assay. From the total miRNAs detected, 2 were upregulated and 25 were downregulated in the *p* < 0.01 group, 32 were upregulated and 90 were downregulated in the *p* < 0.05 group (122 total differentially expressed), and 41 were upregulated and 124 were downregulated in the *p* < 0.1 group (165 total differentially expressed) (Fig. 3c). Of the 269 detected miRNAs, 184 were similar between FA and CAPs exposed groups (Fig. 3d). Two of the most highly expressed miRNAs in the CAPs exposed groups were in the *mmu-let-7a* family, which warranted further in vitro transfection experiments.

### Gene ontology

**Fig. 4:**
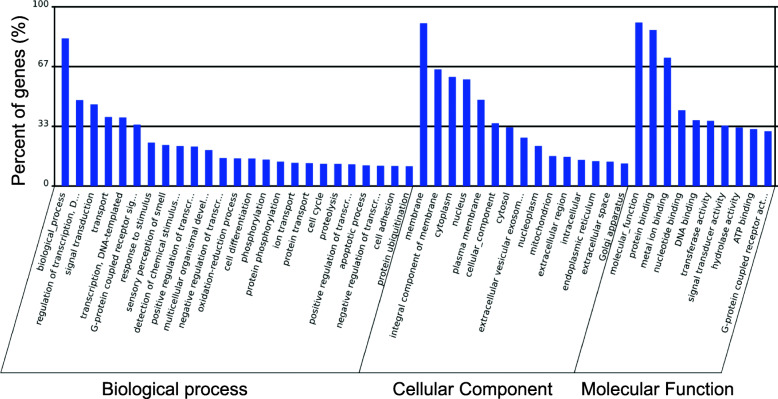
Gene ontology. Representation of gene and gene product attributes via gene ontology (GO, http://www.geneontology.org). Percent genes (%) involved in biological processes, cellular component or molecular function. Significant GO terms were calculated and those GO terms with a *p*-value < 0.05 are defined as significant

**Fig. 5:**
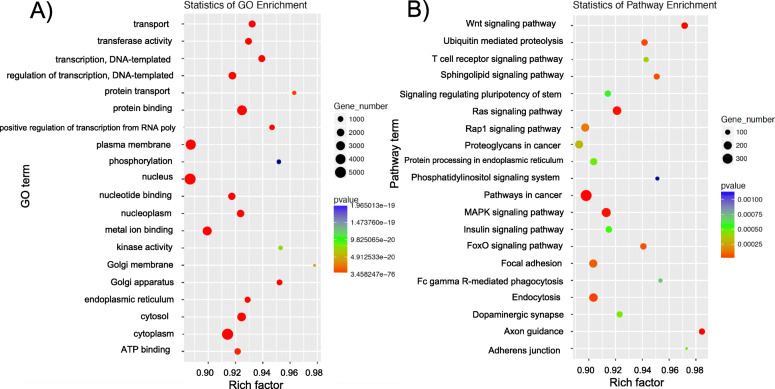
Gene ontology (**a**) and KEGG pathway enrichment (**b**) Gene number is defined as the number of target genes in each term or pathway. The Rich factor is defined as the number of target genes divided by the number of all the genes in each term or pathway. The number of GO target genes, *p*-value and rich factor are indicated in the scatterplot. KEGG (Kyoto Encyclopedia of Genes and Genomes, http://www.kegg.jp) consists of a collection of databases including genomes, biological pathways, diseases, drugs and chemicals. KEGG pathways were calculated and those with a *p*-value < 0.05 are defined as significant

Gene ontology (GO) represents the initiative of gene and gene products. Significant GO terms are listed in Supplemental Table 1. The highest significant percentage of genes involve biological processes, membranes and molecular functions (Fig. 4). The scatterplot of GO Enrichment (Fig. 5a) graphically depicts the rich factor. Rich factor = (number of target genes in the GO)/total number of genes in GO term). The larger the rich factor is the greater the enrichment.

### KEGG analysis

KEGG analysis revealed significant changes between FA and CAPs groups as measured in the SCIP assay (Fig. 5b). A complete list of significantly altered pathways is listed in Supplemental Table 2. The most significantly changed groups involved pathways in cancer, axon guidance and Wnt signaling. Pathways involving adherens junctions, and focal adhesion were also among the most highly altered pathways.

### Serum-borne microRNAs and associations with microRNAs in the endothelium

**Fig. 6:**
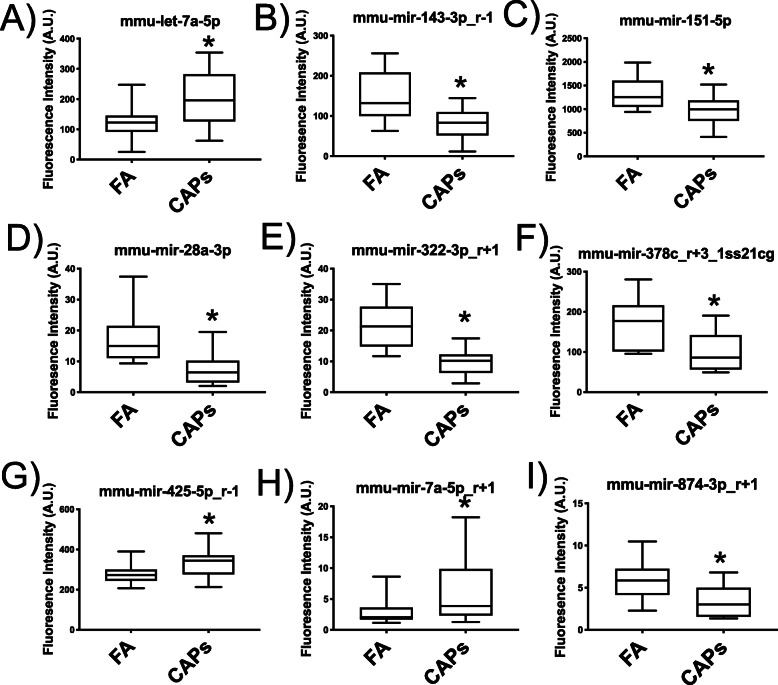
Circulating serum-borne microRNAs Highly significantly differentially expressed miRNAs (*p* < 0.01), as measured in the SCIP assay, were assessed in FA and CAPs serum. **a**-**i** Significantly altered (*p* < 0.05) microRNAs in FA and CAPs-exposed mouse serum

**Fig. 7:**
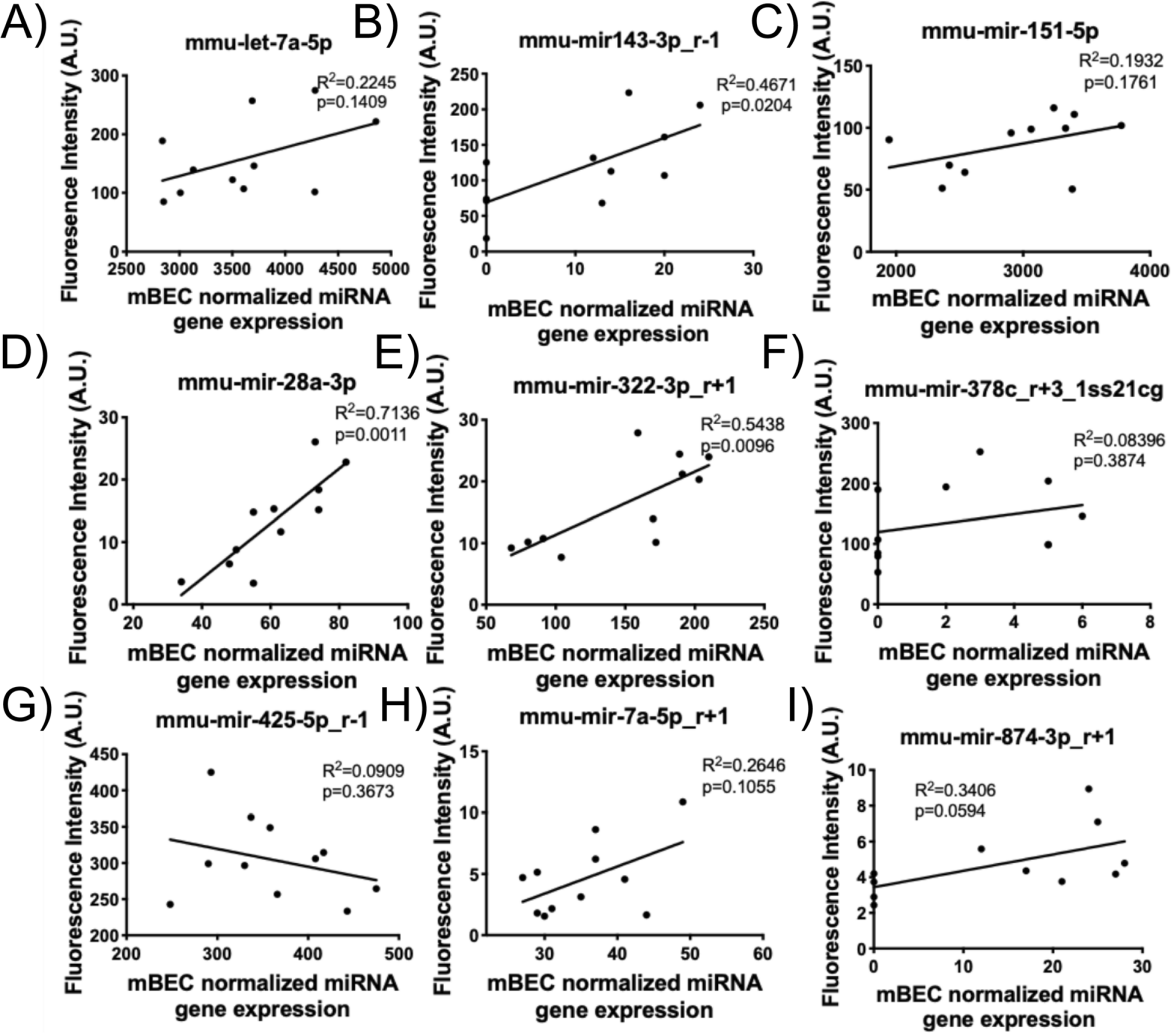
Associations between miRNAs in the mCECs measured in the SCIP assay (x-axis) and serum-borne miRNAs (y-axis). Linear regression analysis reveals significant positively correlated associations between miRNAs in mCECs, as measured in the SCIP assay and serum-borne miRNAs, with the exception of *mmu-mir-425-5p_r-1* (**g**), which demonstrates a negative association between cells and serum

From the 27 murine-origin miRNAs that were analyzed in the serum, 9 were differently expressed (*p* < 0.05). All serum-borne miRNAs, except for one (mmu-mir-425p_r-1)(8/9 miRNAs), demonstrated the same change in expression, either upregulation or downregulation as seen in mCEC miRNAs following SCIP (Fig. 6a-i). Linear regression analysis between the serum-borne miRNAs and mCEC miRNAs as measured in the SCIP assay indicated linear associations among several of the miRNAs, including significant (*p* < 0.05) associations in mmu-mir-143-3p_4–1 (*R*^2^ = 0.4671, *p* = 0.0204), mmu-mir-28a-3p (*R*^2^ = 0.7136, *p* = 0.0011) and mmu-mir-322-3p_r + 1 (*R*^2^ = 0.5438, *p* = 0.0096) (Fig. 7b, d, and e, respectively).

### Target mRNA pathway analysis

**Fig. 8:**
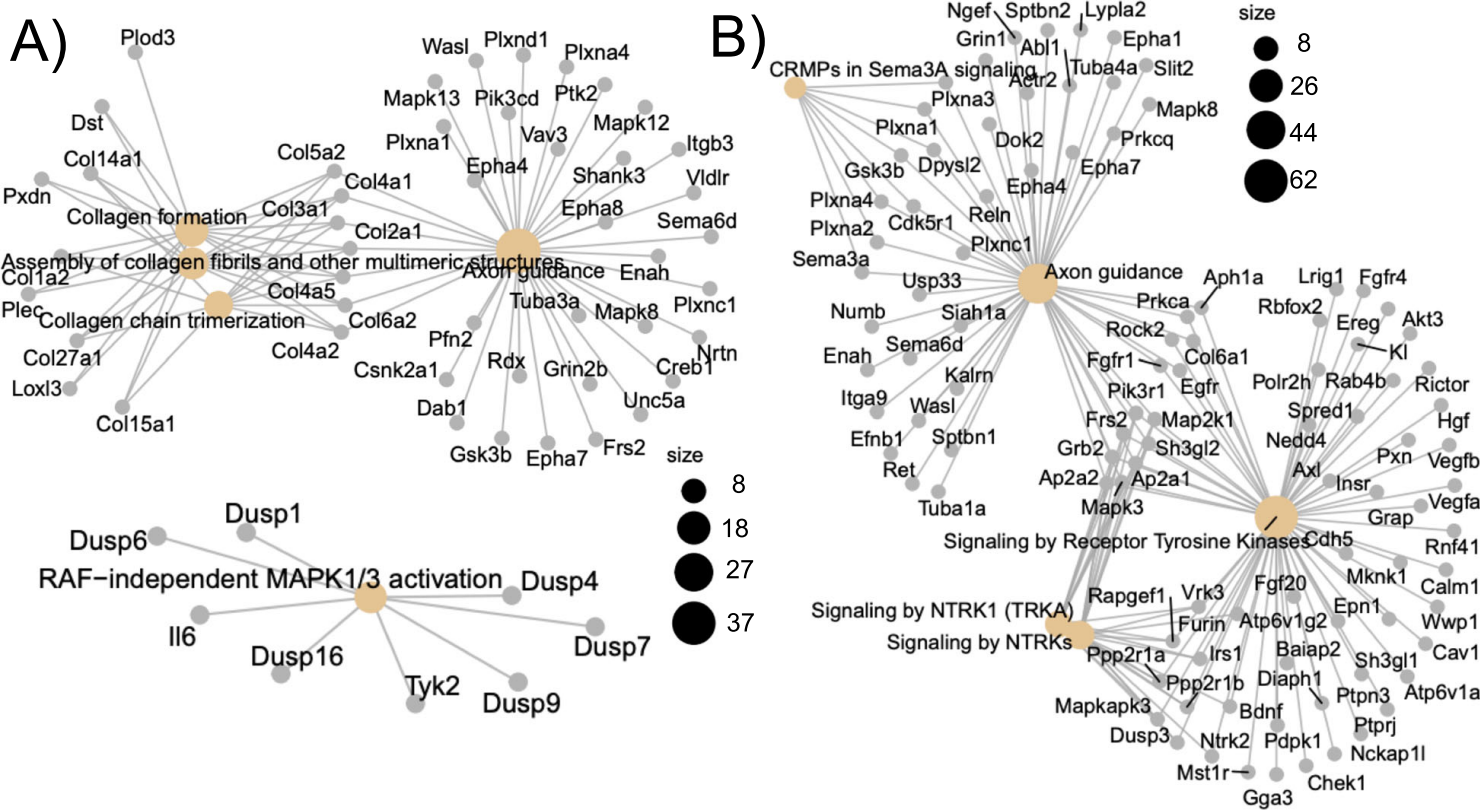
Pathway analysis A) Pathway analysis for upregulated (**a**) and downregulated (**b**) miRNAs and their respective mRNA targets. Upregulated genes involved RAF-independent MAPK activation, the MAPK pathway, axon guidance, assembly of collagen fibrils and other multimeric structures and collagen chain trimerization pathways. Downregulated genes involved the RAF/MAP kinase cascade, axon guidance, collapsing response mediator proteins (CRMPs) in Sema 3A signaling and signaling by receptor tyrosine kinases

Pathway analysis revealed the most significantly upregulated miRNAs (*p* < 0.01) targeted MAP kinase activation, axon guidance, and collagen-related genes (Fig. 8a). The most significantly downregulated genes (*p* < 0.01) affected mRNA targets involving RAF/MAP kinases, signaling by tyrosine kinases, NTRK (neurotrophin receptors), axon guidance and CRMPs (collapsin response mediator protein) in Sema3A signaling (Fig. 8b).

### *Let-7a* overexpression in mCECs

**Fig. 9:**
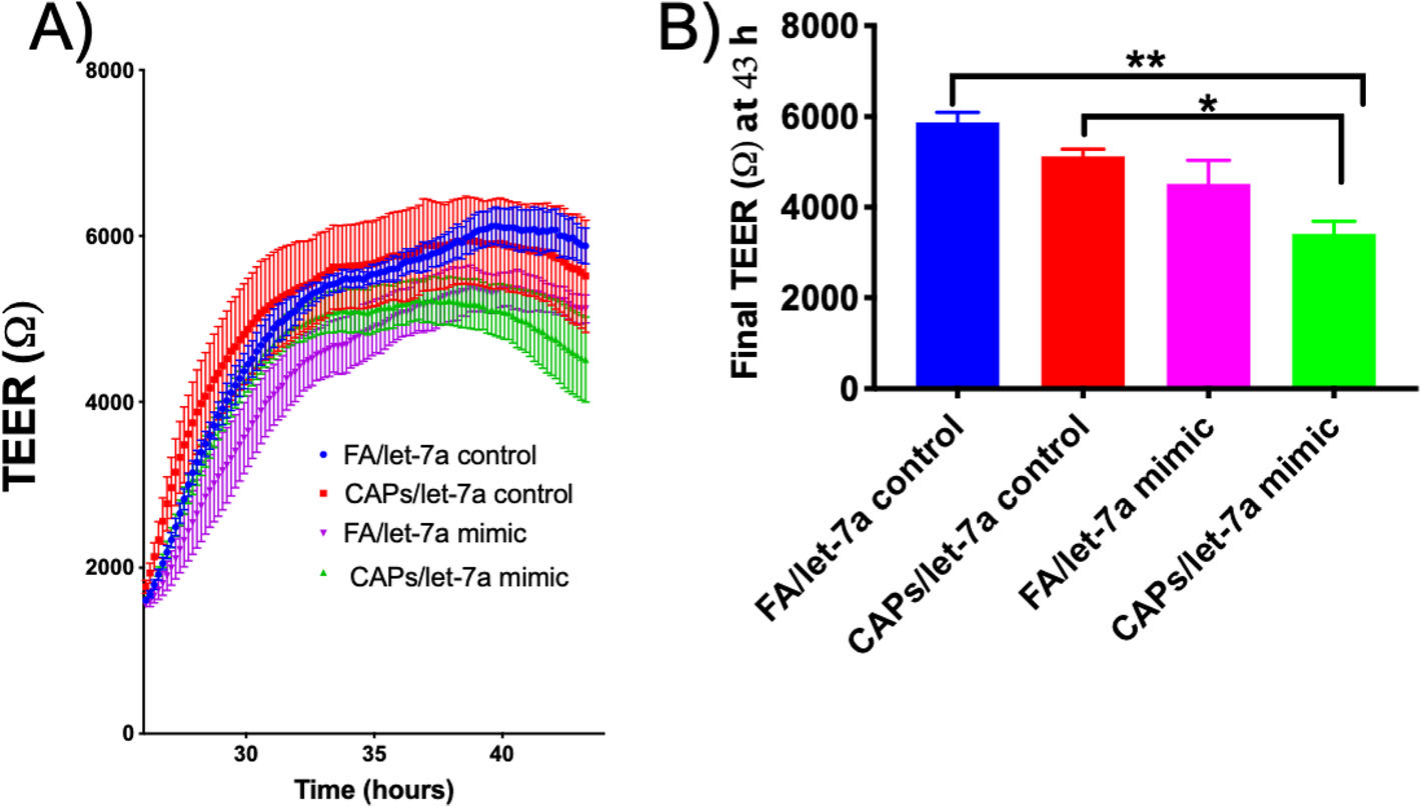
Transendothelial electrical resistance (TEER) mCEC resistance following transfections with *mmu-let-7a* controls and mimics and incubation with serum from FA and CAPs-exposed mice. ANOVA followed by Tukey’s post-hoc test indicates significant differences between groups (*p* < 0.05). **a** mCEC transendothelial resistence over time. **b** final transendothelial resistence at 43 h after curve plateau

*Mmu-let-7a* was significantly upregulated in the CAPs-exposed group, as measured in the SCIP assay (Fig. 3a), which warranted further investigation into this miRNA’s role in the cerebrovascular endothelial cells. Results suggest that overexpression of transfected exogenous *mmu-let-7a* in the presence of serum from CAPs-exposed mice resulted in a significant decrease in mCEC barrier integrity, as determined by transendothelial electrical resistance (TEER, ^, Fig. 9a). Let-7a mimic/CAPs group demonstrated a significant TEER decrease from both the FA/let-7a control and CAPs/let-7a control (Fig. 9b). Results suggest that overexpression of *mmu-let-7a* may decrease mCEC integrity in vitro.

## Discussion

This is the first assessment of mCEC miRNA alterations as a result of serum-borne factors from mice exposed to FA and CAPs. Using a real-time, inhalation toxicology system, we captured concentrated ambient, on-site CAPs near the Claim 28 AUM on Navajo Nation, AZ for inhalation exposure. Because of the uniqueness of our paradigm we have preliminarily assessed neurovascular toxicity, using a novel in vitro assay, as a result of these inhaled exposures.

Due to this improper clean-up, many of the surrounding tribal communities are exposed to varying degrees of contaminant metal mixtures, including uranium (U), arsenic (As) and vanadium (V) [4–6]. Metals-based exposure has been implicated in several adverse health effects, including pulmonary and cardiovascular diseases, neurological disorders and cancer [53–56]. Recent epidemiological evidence from the area has suggested cognitive deficits in Navajo children compared to the national average [57]. We surmise that inhaled exposures from AUMs may play a role in this etiology of neurological outcomes.

We assessed the most significantly differentially expressed miRNAs (*p* < 0.01) between CAPs and FA-exposed mice, as measured in the SCIP assay. In the CAPs exposure group, the most highly upregulated miRNAs of murine origin were in the let-7a family (mmu-let-7a-5p, mmu-miR-7a-5p_R + 1). These data are consistent with epidemiological evidence demonstrating upregulated let-7a in the serum of elderly men exposed to CAPs at the 1-year window [58]. Additionally, acrolein-exposed human umbilical vein endothelial cells exhibited a decrease in endothelial cell migration and insulin sensitivity following induction of let-7a [59]. Let-7a plays a prominent role in cell growth and differentiation [60], which may contribute to cerebrovascular endothelial function. In addition, overexpression of *let-7a* regulates angiogenesis by targeting TGFBR3 and reduces endothelial cell migration rate, in vitro [61]. Other studies from our laboratory have noted BBB leakage following other inhaled exposures such as multi-walled carbon nanotubes [34, 62] and ozone, a common ground-level air pollutant [35]. Similarly, we found that let-7a overexpression using a transfected let-7a mimic resulted in a decrease of transendothelial barrier resistance as measured in the SCIP assay. However, our current data suggest that upregulation of miRNA may play a unique role in endothelial or BBB disruption following inhaled air pollution exposures. In support of our findings are documented associations between metal exposure and circulating miRNAs [29–31]. Air pollution is a mixture of particulates and gases such as O_3_ and NO_2_ and human populations exposed to traffic pollution exhibit alterations in circulating miRNAs following a minimal 2 h exposure [63]. Circulating miRNAs are also biomarkers of radiation-induced cardiac toxicity [64], and low dose occupational exposure to organic solvents was associated with upregulated miRNAs, including miR_6819_5p and miR_6778_5p [65]. Additionally, bioinformatic analysis suggests that circulating miRNAs reflect the adverse toxic consequences in the lung, heart, kidney and brain [32, 33].

One of the most profound insights from this study is the similarity between 89% (8/9) of the miRNAs tested in cells, as measured by the SCIP assay and serum. These miRNAs assessed in the FA or CAPs serum exhibited the same directional change (either upregulation or downregulation) as the cellular miRNAs, as measured in the SCIP assay. This may suggest that certain miRNAs could potentially transfer from the extracellular compartment into the endothelium. In addition to miRNA translocation, other mechanisms may be at play such as extracellular matrices or serum-borne peptide fragments influencing miRNA transcriptional change within the endothelium. Additionally, these key cellular and serum-borne miRNAs, may suggest potential therapeutic targets in populations exposed to high levels of air pollution. Previous literature has suggested that, following inhaled toxicant exposure, peptide fragments are derived from lung circulate and interact with endothelial scavenger receptors, thereby activating downstream targets in the inflammasome including NFkB [17, 34, 62]. Many of the downstream target genes, as determined by pathway analysis, such as ROCK2 and VEGFA and B are involved in actin-cytoskeleton reorganization and/or angiogenesis. This may indicate that these altered miRNAs are thereby responsible for regulating endothelial BBB disruption following inhaled toxicant exposure as previously documented from our laboratory [34, 35]. In support of our findings, previous reports have implicated air pollution in neuroinflammation and diseases of the central nervous system [39–41]. Subchronic exposure to diesel exhaust was shown to elevate markers of early neuropathology such as tau and A42, two markers of Alzheimer’s disease and ⟨ Synuclein, a preclinical biomarker of Parkinson’s disease-like pathology [39]. Additionally, PM has been identified in olfactory bulb neurons following long-term air pollution exposures [66]. Previous studies from our laboratory indicated that Fasudil, a well-established ROCK inhibitor, prevents particulate-induced BBB dysfunction [34]. Our data now suggest that these downregulated miRNAs can control ROCK expression and may eventually serve as potential therapeutic targets in vulnerable, CAPs-exposed populations.

By utilizing the AirCARE1 inhalation system, we anticipate a realistic characterization of the overall contribution of on-site, wind-blown dusts to health outcomes. Although this mobile laboratory is able to capture real-time, in situ CAPs, one limitation of the AirCARE1 exposure paradigm was that no head-to-head comparisons with other regional PM were made, which is a current limitation of this work. In the present study, the concentration levels in the chamber mimicked concentrations found in industrial cities around the world [67, 68]. However, they were still far below concentrations found in dust storms in desert environments [69]. Analytical methods revealed CAPs metal-enrichment including detectable levels of Mo, Cd, Gd, W, Pb, U, Mg, Al, V, Fe, Ni, Cu, Zn, As and Se. While many of these metals have been well-established as drivers of cardiopulmonary toxicity [6, 70, 71], others, including rare earth metals such as Gd have been less characterized as contributors to health effects. Future studies will focus on PM organic composition in the chamber in addition to the inorganic, metals fraction.

One limitation of this study is the in vitro nature of our bioassay and future studies may focus on isolation of brain endothelial cells post-exposure. Another key limitation of the study was the limited time frame for collection/analysis of CAPs relative to wind direction. While we had a reasonable distribution of wind coming from the mine site, we did not note any obvious impact of wind directionality on the levels of uranium or vanadium. Due to technical limitations with the chamber filters, we were also unable to analyze the CAPs organic fraction. Longer exposure campaigns would help to better understand the connection between meteorological factors and resuspension of mine site-derived metal contaminants.

## Conclusions

In the present study, we show that serum from mice exposed to ambient AUM-derived CAPs in the central region of Navajo Nation (Claim 28) induces significant changes in cellular murine miRNA expression as measured in the SCIP assay. Validation of the highly differentially expressed cellular miRNAs in the serum revealed that 30% were significantly altered. Furthermore, 89% of the miRNAs assessed in the serum of FA or CAPs-animals exhibited the same directional change (either upregulation or downregulation) as the cellular miRNAs. These data suggest that miRNAs from systemic circulation may translocate to the endothelium following CAPs exposure. The relative characterization of circulating serum-borne miRNAs and cellular miRNA expression following SCIP may add insight into the most significant potential clinical targets. More importantly, because the AirCARE1 CAPs exposure paradigm captures real-time, on-site world air pollution, these data are also directly impactful and translationally relevant to the Native American communities at risk for mine-site dust exposure. To our knowledge, this is the first study of its kind to suggest that environmentally-driven changes to the circulating/extracellular milieu may be responsible for driving intracellular endothelial miRNA alterations, which may include both upregulated and downregulated miRNAs. Understanding the complex protein/lipid/small molecule changes to the circulation after air pollution exposures will be crucial to understanding the endothelial responses, especially in the context of vascular disease. Further mechanistic research highlighting miRNA trafficking and potential mechanistic miRNA targets is warranted. Future studies should focus on the role of circulating extracellular ligands as drivers of cellular miRNA expression.

## Supplementary information

**Additional file 1.**

**Additional file 2.**

## Data Availability

All data are available upon reasonable request. Bioinformatics and sequencing data are made available through a repository found at https://github.com/Perl-R/190606-Uranium-miRNA.
